# Attitudes on data reuse among internal medicine residents

**DOI:** 10.5195/jmla.2024.1772

**Published:** 2024-04-01

**Authors:** Fred Willie Zametkin LaPolla, Genevieve Milliken, Colleen Gillespie

**Affiliations:** 1 fred.lapolla@nyulangone.org, Research and Data Librarian, Head of Data Services, NYU Health Sciences Library, NYU Langone Health, New York City, NY; 2 Genevieve.Milliken@nyulangone.org, Data Services Librarian, NYU Health Sciences Library, NYU Langone Health, New York City, NY; 3 colleen.gillespie@nyulangone.org, Institute for Innovations in Medical Education, New York University Grossman School of Medicine, NYU Langone Health, New York City, NY

**Keywords:** Graduate Medical Education, data reuse, secondary data analysis, surveys, residents, data services, data curation, GME

## Abstract

**Background:**

NYU Langone Health offers a collaborative research block for PGY3 Primary Care residents that employs a secondary data analysis methodology. As discussions of data reuse and secondary data analysis have grown in the data library literature, we sought to understand what attitudes internal medicine residents at a large urban academic medical center had around secondary data analysis. This case report describes a novel survey on resident attitudes around data sharing.

**Methods:**

We surveyed internal medicine residents in three tracks: Primary Care (PC), Categorical, and Clinician-Investigator (CI) tracks as part of a larger pilot study on implementation of a research block. All three tracks are in our institution's internal medicine program. In discussions with residency directors and the chief resident, the term “secondary data analysis” was chosen over “data reuse” due to this being more familiar to clinicians, but examples were given to define the concept.

**Results:**

We surveyed a population of 162 residents, and 67 residents responded, representing a 41.36% response rate. Strong majorities of residents exhibited positive views of secondary data analysis. Moreover, in our sample, those with exposure to secondary data analysis research opined that secondary data analysis takes less time and is less difficult to conduct compared to the other residents without curricular exposure to secondary analysis.

**Discussion:**

The survey reflects that residents believe secondary data analysis is worthwhile and this highlights opportunities for data librarians. As current residents matriculate into professional roles as clinicians, educators, and researchers, libraries have an opportunity to bolster support for data curation and education.

## BACKGROUND

Secondary data analysis or data reuse is an area of interest to librarians working in data services. Organizations such as the Data Discovery Collaboration (DDC), the Data Curation Network (DCN) and the Research Data Access and Preservation Association (RDAP) work with information professionals to help spread education and infrastructure around data sharing [1–3]. Additionally, the growth of free or no cost-repositories has reduced barriers for storing data for secondary use [4–7]. Research has indicated strong levels of interest in data sharing among scientists across many different disciplines [8–14]. Our study aimed to build upon past work in this area with a novel focus specifically on medical residents' attitudes on secondary data analysis.

NYU Langone Health's Department of General Internal Medicine and Clinical Innovations provides a research block to all Postgraduate Year 3 (PGY3) Primary Care (PC) residents called the Research Practicum. The Research Practicum employs a secondary data analysis methodology, using either nationally-available data identified using our institutional data catalog or locally collected data from researchers at our institution who have allowed instructors to use their data for educational purposes [15]. This focus on secondary data analysis makes the Research Practicum unique in the medical education literature; as such, these residents provide a unique perspective of being General Medical Education (GME) trainees who also have exposure to data reuse [16–43].

Providing research training is important to GME programs for several reasons. The Accreditation Council of Graduate Medical Education (ACGME) explicitly requires that medical residents engage in scholarly activity, though the specifics are broadly written so as to give programs a great deal of leeway as to how that requirement is met [46]. That said, providing meaningful exposure to research is associated with positive outcomes for physicians. For example, Mills et al., Dengel et al., Fancher et al., Macknin et al., and Robertson et. al correlate participation in residency research programs with post-residency publishing and increased likelihood of grant awardees, though it is possible this correlation reflects a selection bias (i.e. that those who are drawn to research as residents continue to be drawn to research after residency) [16,25–26,34,36]. Nevertheless, providing opportunities to explore research at a minimum allows those with an interest in research to explore their passion, while also giving hands on exposure to residents who may be unsure about their desire for a research career.

While many residency programs have well-documented research programs [16–43], there is considerably less examination of how often residents gain experience engaging with secondary data analysis in formal settings. This is despite availability of sources of data for secondary analysis [4–7]. As a note on usage, this paper uses the term secondary data analysis to mean a research methodology using research information collected by others to obtain new insights [44]. In the library and information science literature this concept is often described as “data reuse.” In discussions with residency directors and a chief resident, “secondary data analysis” was strongly preferred as being more familiar than “data reuse.” In keeping with their usage, this paper uses “secondary data analysis,” but we view the two terms as interchangeable.

This research grew out of a larger project aiming to understand if a research practicum style block would work in other residency tracks, specifically Categorical Medicine and a Clinician-Investigator (CI) Track, where currently a secondary data analysis research block is not included. While all three tracks are in our internal medicine (IM) department, Categorical focuses more on inpatient care, PC focuses more on ambulatory care, and CI has greater emphasis on research. This research provides an opportunity to understand the views of IM residents at an urban academic medical center on secondary data analysis.

This research was approved by our institution's IRB, s22-00050.

## CASE PRESENTATION

### Survey Design and Administration

This case report employed a survey methodology of residents in the Department of Internal Medicine and Clinical Innovations (DGIMCI) at NYU Langone Health in the PC, Categorical and CI tracks. All PGY3s in our PC track are exposed to secondary data analysis, in contrast to the other tracks, and for the current analysis we were primarily interested in overall attitudes about secondary data analysis and focused on overall attitudes as one population of DGIMCI residents.

Our secondary data analysis questions were based on questions relating to data reuse from Curty et al's “Attitudes and Norms Affecting Scientists' Data Reuse” and Tenopir et al.'s “Changes in Data Sharing and Data Reuse Practices and Perceptions Among Scientists Worldwide,” as part of a larger pilot study on implementing a research block in residency tracks [8,11]. Surveys were built by modifying existing questionnaires in discussion with residency leaders. For example, we simplified some of the questions based on conversations with residency stakeholders, as we concluded that the original wording may be too abstract in the context of medical residency.

Anonymous surveys were built in our institution's instance of REDCap and can be viewed on this project's OSF page [45]. We distributed the surveys by QR codes on flyers that were distributed in person at residents' meetings. Residents were reimbursed with a $10 gift card, which was done to incentivize their time without being large enough to be coercive. Questionnaires were designed to be completed in five to ten minutes to avoid survey fatigue. While this sacrificed validity and the ability to make direct comparisons to other research, the pragmatic consideration of making a shorter survey that residents were more likely to fill out to completion was given priority.

In our survey, secondary data analysis was defined as:

“conducting new analyses to answer a research question that are separate from the stated research goal of the researchers who collected the data. Examples may include, but are not limited to: Using data from a large study such as the NIH Health Information National Trends Survey, https://hints.cancer.gov/, to answer a new research question; Requesting data from a researcher to conduct analyses separate from their original research question; Downloading data from an online repository to analyze; Applying for access to data from a public agency or research institution to conduct analysis on data they store.”

Using this description, residents were asked to answer on a four-point Likert scale if they believed secondary data analysis is worthwhile and if residents should be trained to do it. For these original questions, we opted for a four point because a four-point scale does not allow respondents to choose a neutral option and forces an overall choice on the part of the study participant [48]. Based on Tenopir et al. [11] residents were then asked to rate on a five-point Likert scale, with a sixth “Unsure” option, if they felt secondary data analysis: saves time, is efficient, is easier than collecting their own data, is hard to explain in a methods section, improves results, helps answer research questions, is harder than conducting research with their own data, and takes longer than conducting research with their own data.

We hypothesized that in general IM residents would be interested in using secondary data analysis methods and specifically that they would believe that it helps save time, is more efficient than collecting original data (as defined by conducting a new study rather than analyzing existing data collected by someone else), is easier than collecting original data, that it would be easy to explain in methods, help improve their own research results and answer research questions, and be easier and faster than conducting research with their own data.

**Figure 1:**
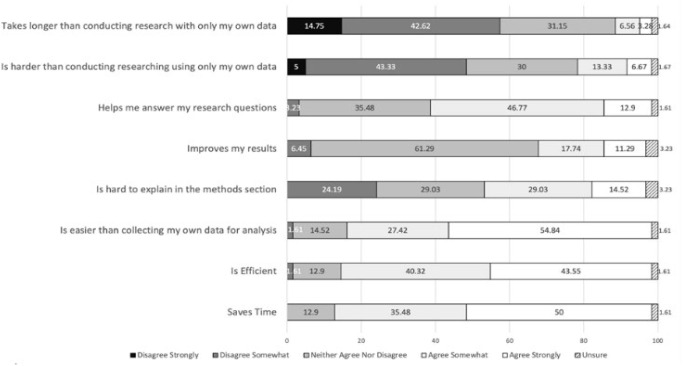
Percentages of residents who feel that each statement relates to secondary data analysis.

Data was analyzed using R version 4.0.3 and RStudio Version 1.3.1093. The image for Figure 1 was made using analysis in R and graphed in Excel version 16.76. Surveys were administered in Spring 2022. Results were also shared with residency directors to help contextualize the data and to confirm that results resonated with their understandings of the residency tracks. We employed the Tidyverse package to help clean and analyze data [47].

### Survey Results

The total population of residents was 162, with 114 coming from the Categorical Track, 18 from Clinician-Investigator Track and 30 from Primary Care. 67 residents completed the survey, marking a completion rate of 41.36%. Of these 12 (18%) were PGY1, 31 (46%) PGY2 and 24 (35%) were PGY3. By track, the completion rate was: Categorical 30.7%, PC 83.3% and CI 38.9% relative to each of their total track sizes. See Table 1 for the Demographics breakdown.

**Table 1:** Demographics of study participants by year, gender, race and ethnicity.

	Number of responses (percentage)
Total number or responses	67
PGY1	12 (18%)
PGY2	31 (46%)
PGY3	24 (35%)
Gender	
Female	24 (44%)
Male	30 (66%)
Race	
White	31 (58.5%)
Black/African American	3 (5.7%)
Asian or Asian American	16 (30.2%)
Other	1 (1.9%)
Ethnicity - Hispanic/Latino	5 (9.3%)

A majority of respondents (88.7%) felt secondary data analysis is worthwhile, with residents in the CI track rating it slightly more highly (median rating of worthwhileness CI: 4, PC: 3, Categorical: 3, p = .03 in a Kruskal Wallis test, where a 4 indicated “strongly yes” and a 3 a “Yes” on a Likert scale). Additionally, 49 (79.0%) out of 62 stated that residents should be trained in secondary data analysis, and this was consistent across tracks as demonstrated by a non-significant Kruskal-Wallis test highlighting no difference between groups (p = 0.36). Results were consistent across tracks with the exception that Categorical residents were slightly less likely to find secondary data analysis efficient (Kruskal-Wallis test p = 0.02, median score 4 vs 5 for other groups), time saving (Kruskal-Wallis test p = 0.01, median score 4 vs 5 for other groups) or to be useful for answering their own research questions (Kruskal-Wallis test p = 0.01, median score 3 vs 4 for other groups).

Most residents surveyed (80%) indicated that they believe secondary data analysis saves time, is efficient and is easier than collecting original data, as determined by agreeing somewhat or strongly with statements on secondary data analysis vs not. Notably, residents were divided on the question of if secondary data analysis was more difficult to explain in methods sections and if it could help them answer their research questions. A majority of residents surveyed did not believe secondary data analysis helps them to improve their own research results. See Figure 1 for a breakdown of percentage answers to each question regarding secondary data analysis with all tracks pooled. Residents agree with statements that secondary data analysis is efficient and saves time, but were split on how helpful it is at answering their own research questions of the ease of describing secondary data analysis in a methods section.

## DISCUSSION

The results of this survey highlight opportunities and challenges for librarians working in data services and educators in GME. Notably, strong majorities of residents felt that secondary data analysis is worthwhile and an efficient, time saving method of research. Additionally, residents expressed interest in being trained in this methodology. A majority of residents (59.67%) felt secondary data analysis helps them answer their research questions, highlighting its utility as a methodology for residents. Nevertheless, nearly 61.9% of residents neither agreed nor disagreed that secondary data analysis would help improve their own research results, highlighting a need for access to and education on the use of relevant data sources that can meet the diverse needs and research interests of clinicians. While organizations like the DDC, DCN, and RDAP are doing essential work in this field, further progress may depend on data librarians leveraging their resources and understanding to facilitate data discovery and research infrastructure at their institutions [1–3]. For example, a potential area for future librarian skill development may be to learn to provide assistance in identifying usable data specific to researchers' needs.

Residents were less clear in their views on how easy it is to explain secondary data analysis methods in a paper, which highlights an opportunity for educators working in data reuse and secondary data analysis. 45% of residents opined it would be hard to explain their work in methods sections. By comparison, 75% of respondents in the work done by Tenopir et al. shared concerns about misrepresentations due to complexity of data [11]. In other words, residents and research scientists alike are both very concerned about communication and comprehension of their work. The concern about complexity and communication highlights an opportunity for those employed in GME research and data education, namely to help explain the processes of secondary data analysis, and how to compose a methods section employing this methodology, as well as explaining issues around data citation.

Finally, it stands to reason as residents move into roles as clinicians, educators and researchers, the interests and views they hold today may shape clinical research in the future. If residents value secondary data analysis, then it is incumbent on libraries and research institutions to invest in data curation and data infrastructure, as well as for GME programs to consider incorporating these skills into their training. Investment in data infrastructure can include investing in data catalogs and expertise in data discovery, but will also need to be paired with training in analysis and how to work with data once obtained to avoid biased results. For example, in our experience having librarians with data curation expertise has allowed us to identify data sources that can meet residents' interest and be incorporated into projects that are meaningful to learners, but an ongoing challenge remains having scalable ability to instruct residents in its use beyond a relatively small group. Specifically, at NYU Langone Health the Research Practicum relies on the use of our institutional Data Catalog, which is maintained by the library, highlighting the benefit of investing data infrastructure. Should a similar curriculum be of broader interest in the future it stands to reason that other institutions may also have an interest in developing data infrastructure.

Fortunately, because of work being done by data curation institutions, individual librarians may not be ‘on their own' in developing services that can leverage local datasets, and instead they can work with national organizations to gain guidance on how to curate and aid in data discovery locally. For example, the Data Curation Network creates educational programs and working groups to provide individual curators with training and a community of practice as well as connecting institutions for collaboration [3]. Similarly, the Data Discovery Collaboration has allowed 11 institutions to collaborate and exchange information around data cataloging, metadata and outreach strategies in data curation [1]. We hope that as secondary data analysis becomes more common, so too will training opportunities for librarians and GME educators in how to work with the data being curated for reuse.

## LIMITATIONS

This study featured residents from a single program in one academic medical center, and results at other locations and with different subjects may be different. Additionally, our survey instrument is not validated. As such, results cannot be generalized.

In our results PC is relatively over-indexed, and we speculate this may be due to active librarian teaching roles in the track leading to more willingness on the part of residents to spend time filling out surveys for someone they have a relationship with. This highlights an additional possible area of bias: that those participating may be self-selecting to be those who are highly engaged or have an interpersonal desire to assist in the research, limiting how representative they may be.

Residents also tend to have less research experience and exposure than the broader body of researchers. As such, their expressing that describing secondary data analysis in methods sections may indicate less familiarity and ease with scientific writing overall.

## DATA AVAILABILITY STATEMENT

Anonymized data and questionnaires associated with this study are available on OSF: https://osf.io/n28yj/.

## AUTHOR CONTRIBUTIONS

Fred LaPolla: conceptualization, data curation, formal analysis, funding acquisition, investigation, methodology, project administration, resources, software, visualization, writing – original draft, review, and editing. Genevieve Milliken: conceptualization, investigation, writing – review and editing. Colleen Gillespie: conceptualization, funding acquisition, methodology, writing – review and editing.
